# Supplementary material for: The Evolution and Characterization of the RNA Interference Pathways in Lophotrochozoa
Source: Genome Biol Evol. 2024 May 7;16(5):evae098. doi: 10.1093/gbe/evae098 (PMC11114477; doi:10.1093/gbe/evae098)
Supplement: evae098_Supplementary_Data [file evae098_supplementary_data.zip › Supplementary_materials.zip!/selected_miRNAs/NoGe_selected_miRNAs/mirdeep_pdf_files/NMRB01000004.1_Notospermus_geniculatus_strain_Ushimado_scaffold4,_whole_genome_shotgun_sequence_160.pdf]

Provisional ID	:	NMRB01000004.1_Notospermus_geniculatus_strain_Ushimado_scaffold4,_whole_genome_shotgun_sequence_160
Score total	:	81.1
Score for star read(s)	:	3.9
Score for read counts	:	76.1
Score for mfe	:	1.7
Score for randfold	:	0
Score for cons. seed	:	-0.6
Total read count	:	161
Mature read count	:	107
Loop read count	:	0
Star read count	:	54

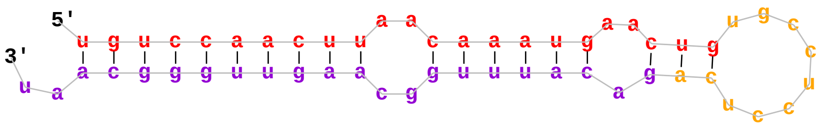

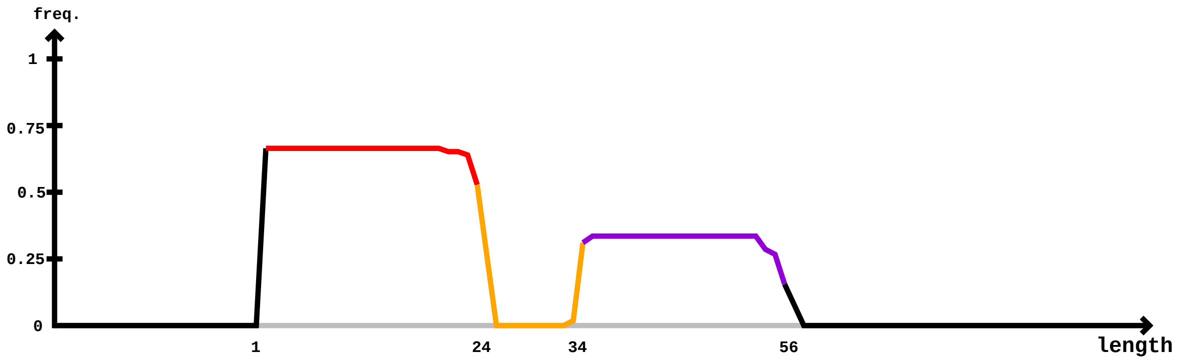


```
                     Mature                               Star
5'- ggcggcuuggcuggagcaguuguccaacuuaacaaaugaacugugccuccucagacauuuggcaaguugggcaauauguuucagacaucaaaggucuauguguuccuuugc   -3'    obs
    ggcggcuuggcuggagcaguuguccaacuuaacaaaugaacugugccuccucagacauuuggcaaguugggcaauauguuucagacaucaaaggucuauguguuccuuugc          exp
    (((.(.((((((((((((((((((((((((..((((((..(((........))).))))))..)))))))))))).))))))))...)))).).)))..............    reads    mm    sample
    ....................uguccaacuuaacaaaugaacu.....................................................................    7        0     Fm3
    ....................uguccaacuuaacaaaugaacug....................................................................    26       0     Fm3
    ....................................................agacauuuggcaaguugggcGaua...................................    2        1     Fm3
    .....................................................gacauuuggcaaguugggc.......................................    6        0     Fm3
    .....................................................gacauuuggcaaguugggca......................................    3        0     Fm3
    .....................................................gacauuuggcGaguugggcaa.....................................    1        1     Fm3
    .....................................................gacauuuggcaaguugggcaa.....................................    16       0     Fm3
    .....................................................gacauuuggcaaguugggcaau....................................    4        0     Fm3
    .....................................................gacauuuggcaaguugggcaaG....................................    1        1     Fm3
    ......................................................acauuuggcaaguugggcaa.....................................    1        0     Fm3

    ....................uguccaacuuaacaaauga........................................................................    1        0     Mm3
    ....................uguccaacuuaacaaaugaac......................................................................    1        0     Mm3
    ....................uguccaacuuaacaaaugaacu.....................................................................    1        0     Mm3
    ....................uguccaacuuaacaaaugaacugu...................................................................    1        0     Mm3
    ....................uguccaacuuGacaaaugaacugu...................................................................    1        1     Mm3
    ....................uguccaacuuaacaaauCaacugu...................................................................    1        1     Mm3
    ....................................................agacauuuggcaaguugggc.......................................    1        0     Mm3

    ....................uguccaacuuaacaaaugaacug....................................................................    4        0     Fm1

    ....................uguccaacuuaacaaaugaacugu...................................................................    2        0     Fm2
    ......................................................acauuuggcaaguugggcGaua...................................    3        1     Fm2

    ....................uguccaacuuaacaaaugaacug....................................................................    18       0     Mm1
    .....................................................gacauuuggcaaguuggCcaau....................................    1        1     Mm1
    .....................................................gacauuuggcaaguugggcaau....................................    14       0     Mm1

    ....................uguccaacuuaacaaauga........................................................................    1        0     Mm2
    ....................uguccaacuuaacaaaugaac......................................................................    1        0     Mm2
    ....................uguccaacuuaacaaaGgaacu.....................................................................    1        1     Mm2
    ....................uguccaacuuaacaaaugaacu.....................................................................    9        0     Mm2
    ....................uguccaacuuaacaaaugaacug....................................................................    29       0     Mm2
```

```
                    Mature                           Star
ggcggcuuggcuggagcaguuguccaacuuaacaaaugaacugugccuccucagacauuuggcaaguugggcaauauguuucagacaucaaauggucuauguguuccuuugc
....................uguccaacuuaacaaaugaacugu....................................................................    3    0    Mm2
.....................................................gacauuuggcaaguugggc........................................    1    0    Mm2
```
